# Evaluation of NAG, NGAL, and KIM-1 as Prognostic Markers of the Initial Evolution of Kidney Transplantation

**DOI:** 10.3390/diagnostics13111843

**Published:** 2023-05-25

**Authors:** Guadalupe Tabernero, Moisés Pescador, Elena Ruiz Ferreras, Ana I. Morales, Marta Prieto

**Affiliations:** 1 Toxicology Unit, Universidad de Salamanca, 37007 Salamanca, Spain; g_tafe@hotmail.com (G.T.); moises@usal.es (M.P.); martapv@usal.es (M.P.); 2 Department of Nephrology, University Hospital, 37007 Salamanca, Spain; eruiz_burg@hotmail.com; 3 Institute of Biomedical Research of Salamanca (IBSAL), 37007 Salamanca, Spain; 4 Group of Translational Research on Renal and Cardiovascular Diseases (TRECARD), 37007 Salamanca, Spain; 5 RICORS2040-Instituto de Salud Carlos III, 28029 Madrid, Spain

**Keywords:** kidney transplantation, urinary biomarkers, NAG, NGAL, KIM-1, prognosis

## Abstract

Kidney transplantation is the best option for end-stage chronic kidney disease. Transplant viability is conditioned by drugs’ nephrotoxicity, ischemia–reperfusion damage, or acute rejection. An approach to improve graft survival is the identification of post-transplant renal function prognostic biomarkers. Our objective was to study three early kidney damage biomarkers (N-acetyl-d-glucosaminidase, NAG; neutrophil gelatinase-associated lipocalin, NGAL; and kidney injury molecule-1, KIM-1) in the initial period after transplantation and to identify possible correlations with main complications. We analysed those biomarkers in urine samples from 70 kidney transplant patients. Samples were taken on days 1, 3, 5, and 7 after intervention, as well as on the day that renal function stabilised (based on serum creatinine). During the first week after transplant, renal function improved based on serum creatinine evolution. However, increasing levels of biomarkers at different times during that first week could indicate tubular damage or other renal pathology. A relationship was found between NGAL values in the first week after transplantation and delayed graft function. In addition, higher NAG and NGAL, and lower KIM-1 values predicted a longer renal function stabilisation time. Therefore, urinary NAG, NGAL, and KIM-1 could constitute a predictive tool for kidney transplant complications, contributing to improve graft survival rates.

## 1. Introduction

Kidney transplantation has been a great medical milestone that has increased the life expectancy of patients with end-stage chronic kidney disease (CKD). For this reason, it is the best option for renal replacement therapy (RRT), and it also contributes to reduce the high socioeconomic costs associated with dialysis [1,2].

Between the main factors that affect kidney viability after transplantation are the immunosuppressive drugs. Calcineurin inhibitors (cyclosporine A and tacrolimus) are included in this group of drugs. Their nephrotoxic effects are dose dependent and show great heterogeneity between individuals and insufficient correlation with immunosuppressant plasma levels [3,4,5,6]. Kidney graft is also susceptible to the nephrotoxic effect of other drugs (non-steroidal anti-inflammatory drugs [NSAIDs], aminoglycosides, amphotericin B, etc.) and contrast media, which sometimes could be used in the transplant patient [7,8].

Post-transplant kidney damage can also occur due to acute tubular necrosis (ATN), which clinically manifests as delayed graft function (DGF) and requires RRT during the first week post-transplant [9]. ATN can be produced by inherent renal ischemia–reperfusion to transplantation (organ extraction and preservation processes), and it can be exacerbated depending on donor hemodynamic status [10]. In addition, immunological factors make grafts more susceptible to ischemia–reperfusion injury (higher incidence of ATN in retransplanted and hypersensitized patients) [11,12].

Other causes of impaired renal function after transplantation are acute rejection (AR), graft vascular complications (such as renal artery thrombosis or stenosis), urinary tract obstruction, pyelonephritis, cytomegalovirus or polyomavirus infections, or underlying disease recurrence [13].

The existence or co-existence of the aforementioned factors make the patient particularly susceptible to the graft viability. In these circumstances, one of the most effective tools would be early diagnosis. In clinical practice, monitoring of transplanted kidney function is based on the determination of serum creatinine. However, it has an important limitation: when an increase is observed, more than 70% of the kidney function has been lost [14]. Furthermore, increased serum creatinine is a glomerular filtration marker, but not a tubular damage marker [15]. Another diagnostic tool to determine the damage caused is the kidney graft biopsy but it is an invasive procedure with potential morbidity and mortality, and therefore it has its limitations [16]. For these reasons, there is great interest in identifying new non-invasive markers that show the different causes of dysfunction in renal transplantation with greater precision and can facilitate an early and personalised diagnosis of each patient [17,18]. In this way, the appropriate corrective actions could be implemented to improve transplantation results in short and long term.

In recent decades, some urinary biomarkers have been developed, even clinically validated, to diagnose acute kidney injury (AKI) in a more sensitive, precise, and early manner than serum creatinine or glomerular filtration (which is largely based on serum creatinine) [15,19]. These biomarkers are present in urine because of kidney damage, which triggers a cascade of responses in renal tubular cells, thus stimulating the production and accumulation of certain proteins, generally of low molecular weight, in these cells. These proteins are finally released into urine or into systemic circulation. In the latter case, they can subsequently leak into renal glomeruli, so their destination would also be urine. Some of these proteins have been identified thanks to advanced techniques, such as proteomics, allowing their use as biomarkers for kidney damage diagnosis [15]. These proteins include N-acetyl-d-glucosaminidase (NAG) [14,20], neutrophil gelatinase-associated lipocalin (NGAL) [14,21,22,23], and kidney injury molecule-1 (KIM-1) [24,25,26].

Our objectives in this work were (i) to study the post-kidney transplant evolution of the urinary biomarkers NAG, NGAL, and KIM-1 in relation to the renal function evolution (determined by serum creatinine); and (ii) to evaluate the capacity of these biomarkers to predict possible complications in the initial evolution of kidney transplantation.

## 2. Materials and Methods

### 2.1. Study Design

**Figure 1:**
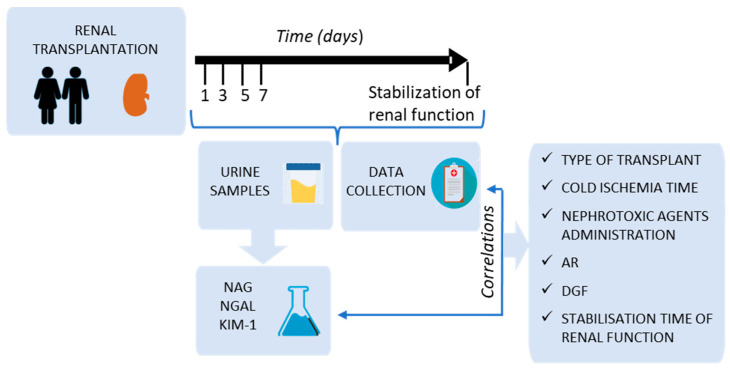
Study design. AR: acute rejection; DGF: delayed graft function.

A prospective longitudinal study was proposed in which patients who were to receive a kidney transplant at the University Hospital (Salamanca, Spain) were included (Figure 1). The protocol was approved by the Clinical Research Ethics Committee of the Salamanca’s Health Area. This protocol did not alter at any time the standard procedure of patient’s medical care. All included patients signed an informed consent, in accordance with the Declaration of Helsinki [27].

The inclusion criteria were the following: patients older than 18 years who underwent surgery to receive a kidney transplant, or a double kidney and pancreas transplant, from a living or cadaveric donor. The only exclusion criterion was that the patients did not agree to participate in the study (patients who did not sign the informed consent).

The patient inclusion period was between July 2013 and January 2015, inclusively. The follow-up time was 3 months from the transplant.

### 2.2. Study Variables

Concentration and urinary excretion of NAG, NGAL, and KIM-1 (all of them early renal damage biomarkers) were analysed in the post-transplant first week (days 1, 3, 5, and 7 after intervention), as well as on the day on which renal function was considered to have stabilised in each patient (according to clinical judgment and based on serum creatinine). Evolution of urinary biomarkers was compared with the evolution of serum creatinine. In addition, associations of these markers with some post-transplant factors or complications were studied: type of transplant (cadaveric, double transplant cadaveric, or living donor), cold ischemia time of the graft, nephrotoxic agents’ administration, AR, DGF, and stabilisation time in post-transplant renal function (Figure 1).

### 2.3. Patient Data Collection

The following data were collected from each patient (Figure 1): age, sex, CKD cause, type of transplant, type of immunosuppressive treatment, cold ischemia time, number of transplants, nephrotoxic drug administration, AR (confirmed by biopsy), DGF, and stabilisation time in post-transplant renal function. In addition, at the same time, the urine samples were collected (see next section), the following parameters were extracted from the patients’ clinical record: serum creatinine and urea, glomerular filtration rate (CKD-EPI), proteinuria, and urinary flow.

### 2.4. Collection and Processing of Urine Samples

From the excreted urine for 24 h, 10 mL samples were collected on days 1, 3, 5, and 7 after transplantation; subsequently, once a week for 3 months. The samples were processed and stored in the Samples Biobank of University Hospital (Salamanca, Spain). Once in the Biobank, the urine samples were centrifuged (2000× *g*, 4 °C, 8 min) to eliminate possible sediments. Subsequently, they were frozen in several aliquots that were stored at −80 °C until use.

### 2.5. Analysis of Urine Samples 

Analytical determinations of NAG, NGAL, and KIM-1 (Figure 1) were carried out in the labs of the Group of Translational Research in Renal and Cardiovascular Diseases (TRECARD), which is part of the Institute of Biomedical Research of Salamanca (IBSAL). In all cases, samples were tested within 6 months of collection. Commercial kits were used, and the manufacturer’s instructions were followed. For NAG, Diazyme™ colorimetric kit (Poway, CA, USA) was used. In the case of NGAL and KIM-1, ELISA kits from BioPorto Diagnostics (Hellerup, Denmark) and Enzo Life Sciences (Lase, Switzerland) were used, respectively. To calculate the daily excretion of biomarkers, the urinary concentration of each marker was multiplied by the corresponding urinary flow rate.

### 2.6. Statistical Analysis

SPSS Statistics (version 20) for Windows program was used. Quantitative variables were expressed as mean plus/minus standard error. Qualitative variables were expressed in patients’ percentages and in absolute numbers. To analyse the relationship between the biomarkers and the different events that occur in the immediate post-transplant period, statistical analyses were performed with the χ^2^ correlation, creating contingency tables. The 70th percentile and *p* < 0.05 were used to consider the values statistically significant. 

## 3. Results

### 3.1. Patient Characteristics and Type of Transplant

Seventy patients who had received cadaveric, double renal–pancreatic cadaveric, or living donor transplants were recruited for the study. In addition, cadaveric transplants were divided into two groups: donors aged 55 or older, and under than 55. This division is based on the different protocol applied in our hospital (University Hospital, Salamanca, Spain) for transplant patients with donors over 55 years of age, in whom a delayed introduction of the anticalcineurin inhibitor was performed. Patient numbers in each group, age, and sex of both donors and recipients, as well as the cold ischemia time, are shown in Table 1.

For cadaveric donors, the most common cause of death was stroke (68%), followed by traumatic brain injury (25%). Other causes of death were asphyxia, drowning, and aortic dissection.

Regarding recipients, CKD causes were very diverse. The most common were diabetic nephropathy (29%), glomerular diseases (22%), and vascular pathology (including malignant arterial hypertension and nephroangiosclerosis) (15%). Other causes were unknown CKD, autosomal dominant polycystic hepatorenal disease, interstitial nephropathies, Alport syndrome, urological pathology, and hemolytic uremic syndrome.

Immunosuppressive therapy was based on the administration of the following drugs: basiliximab or thymoglobuline (induction), tacrolimus, mycophenolate mofetil, and corticosteroids (Table 2).

Living-donor transplant recipients began taking the medication (tacrolimus and mycophenolate mofetil) three days before intervention. Day 0 is considered the day of transplantation.

Incidence of the main post-transplant complications (AR and DGF) are shown in Table 1. Twelve patients presented AR (nine acute cellular rejection and three acute humoral rejection). Thirty-three percent of patients developed DGF, defining it as the need for RRT (hemodialysis) during the first week after transplantation. Among the 70 patients, 10 presented both DGF and RA; 45 patients did not present any of these two complications.

Finally, 19% of transplant recipients received some nephrotoxic agent (not immunosuppressive drugs) (Table 1), such as antibiotics (gentamicin), iodised contrast, or antiviral drugs (aciclovir, ganciclovir, or valaciclovir).

### 3.2. Evolution of the Urinary Biomarkers (NAG, NGAL and KIM-1) and Serum Creatinine after Transplantation

**Figure 2:**
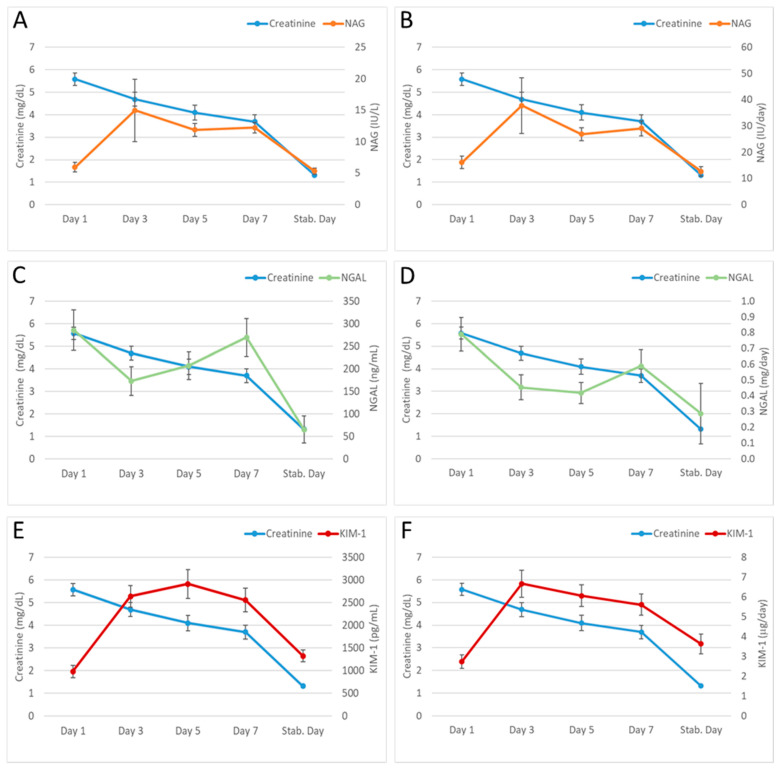
Evolution of serum creatinine, NAG (**A**,**B**), NGAL (**C**,**D**), and KIM-1 (**E**,**F**) on days 1, 3, 5, and 7 after transplant, as well as on the day of renal function stabilisation (day on which serum creatinine had stabilised in each patient). Urinary biomarkers are expressed as urinary concentration (**A**,**C**,**E**) and as daily excretion (**B**,**D**,**F**). Stab. Day: serum creatinine stabilisation day.

The mean of the markers in the set of patients was calculated to compare the urinary biomarker evolution with serum creatinine evolution on the established days (see Section 2.2) after transplantation. Urinary biomarkers levels were expressed both in concentration and in daily excretion (Figure 2) to observe the influence of diuresis.

Figure 2 shows a progressive decrease in creatinine values, until reaching the day of stabilisation. However, the same pattern is not observed in urinary biomarker evolution: NAG and KIM-1 rise on day 3, and they are maintained during the first week, to return to the initial values on the day that creatinine stabilised. In the case of NGAL, a decrease in its levels was observed in the first post-transplantation days; however, it subsequently rises, with a concentration/excretion peak on day 7. Unlike NAG and KIM-1, NGAL levels on the day of creatinine stabilisation are considerably lower than the levels on day 1. Lastly, no significant differences were observed between urinary concentration (Figure 2A,C,E) and urinary excretion of biomarkers (Figure 2B,D,F). No significant differences were found between the established transplant groups: cadaveric kidney transplant (donors aged 55 years or older, or less than 55 years), living donor, or double renal and pancreatic transplant.

### 3.3. Relationship between Urinary Biomarkers (NAG, NGAL, and KIM-1) and the Main Events in the Immediate Post-Transplantation

Urinary biomarker concentrations (i) at the highest point of the first week after transplantation and (ii) on the day on which each biomarker showed an increasing trend (according to Figure 2), were related to: (a) type of transplant (according to the four previously established groups), (b) AR (confirmed by biopsy), (c) DGR, (d) simultaneous AR and DGF, (e) cold ischemia time of the transplanted kidney, (f) administration of nephrotoxic drugs and agents, and (g) time to stabilisation of renal function after transplantation (Table 3).

When analysing contingency tables obtained by Pearson’s χ^2^ test (Supplementary Material), it is observed that patients who received pancreas and kidney transplants had NAG values higher than expected on day 3 after transplant. Moreover, patients were classified into three groups based on cold ischemia time of the graft: low (less than 120 min), intermediate (120–900 min), and high time (more than 900 min). The corresponding contingency table shows that patients (grafts) with an intermediate ischemia time had NAG values higher than expected. Finally, to analyse the relationship of urinary biomarkers with the stabilisation time of renal function, different ranges were established: 30–60 days, 60–90 days, and 90–120 days. The contingency table indicates that patients with a longer stabilisation time have higher NAG values in the first week after transplant.

In relation to contingency tables of NGAL (Supplementary Material), we observed higher values on the seventh day after transplant in patients who received a graft from a cadaveric donor older than 55 years. Moreover, NGAL levels, both on day 7 and in the first week were higher in patients who suffered from DGF. Therefore, there is a greater probability of DGF in patients with elevated NGAL in the first days after transplantation. Similarly, there is a significant statistical relationship between high levels of NGAL in the first week after transplant and the simultaneous appearance of DGFI and AR. The statistical significance is even greater when NGAL values of day 7 are taken, instead of the highest value within the first week. Finally, and as occurred with NAG, patients with a longer stabilisation time (90–120 days after transplant) had higher NGAL values in the first week after transplant.

Regarding KIM-1, contingency tables (Supplementary Material) indicate that patients who received a transplant from cadaveric donors under 55 years of age presented higher values of this biomarker on day 3 after transplant, than the values presented in the other established groups (cadaveric donors over 55 years old, living donors, and double kidney–pancreas transplant). Similarly, the relationship is statistically significant if the highest values for each patient within the first week after transplant are considered. In addition, KIM-1 values appeared to be higher in patients whose graft underwent an intermediate cold ischemia time (120–900 min). Finally, and unlike what occurred with NAG and NGAL, patients who presented a shorter stabilisation time of renal function (30–60 days after transplantation) had the highest KIM-1 values in the first week after transplant.

## 4. Discussion

Urinary biomarkers represent an advance in the early diagnosis of kidney damage and its evaluation, due to this, they offer the possibility of distinguishing between various types and etiologies of AKI [14,28,29]. In this work we have studied, in the context of kidney transplantation, the usefulness of three specific urinary biomarkers of kidney injury, NAG, NGAL, and KIM-1. All of them are classified as early biomarkers since they can be elevated before serum creatinine in some clinical scenarios [30]. The predictive utility of these biomarkers has already been described, both in early detection and in differentiating the nature and severity of lesions, providing prognostic information in the disease course [31,32,33].

Characteristics of patients included in the study (Table 1) largely reflect the current situation of kidney transplantation in terms of CKD causes, donors death causes, or the average age of donors and receivers among others [34,35]. Similarly, descriptive results (AR and DGF prevalence) are also similar to those of other previously published studies [36].

As described in the Results section, four groups of patients were considered: cadaveric donor transplant (older or younger than 55 years), living transplant, and double kidney–pancreas transplant. Groups differ in the immunosuppression regimen received (Table 2), which could influence the transplant evolution due to toxic effects derived from these treatments. On the one hand, anticalcineurin inhibitor introduction was not performed until day 4 after transplant in the case of cadaveric donors over 55 years old, to avoid any possible nephrotoxic effect. On the other hand, patients with a living donor transplant began immunosuppression 3 days before intervention, while those who received a double transplant received thymoglobulin instead of basiliximab as induction treatment. In any case, most of the included patients were in a safe range of anticalcineurin inhibitors level.

To study the biomarker (NAG, NGAL, and KIM-1) evolution after transplantation, their progression was compared with that of serum creatinine during the first week post-transplantation and at the time of renal function stabilisation (Figure 2). While serum creatinine presents a decreasing evolution in the time studied, urinary biomarkers rise at different times. This fact raises the possibility that, although there appears to be an improvement in renal function due to a decrease in creatinine, there is tubular damage or another pathology in the kidney that could determine the medium/long-term prognosis of renal graft [37]. In addition, creatinine evolution in the first days after transplant can be misleading in patients with DGF, since plasmatic concentration can drop due to the cleansing effect of dialysis. In this context, the markers could predict with their increase the individual susceptibility to post-transplant complications such as DGF or AR. According to the literature, NAG elevation on day 3 after transplant could be related to proximal tubule damage, nephrotoxicity, glomerular pathology, papillary damage, or obstructive pathology [38]. NGAL elevation on day 7 post-transplant could be related to nephrotoxicity or inflammation [14,39]. This result is not consistent with those found in other studies [40,41], possibly due to the different inclusion/exclusion criteria carried out. Finally, the KIM-1 increase observed from day 3 after transplant could indicate a failure in proximal tubule regulation, or ischemic/nephrotoxic kidney damage [42].

It is important to note that at renal function stabilisation time (based on serum creatinine), NGAL presents lower values than those found on day 1 after transplant, while NAG and KIM-1 present similar values to the initials (day 1). Data from our lab indicate that levels of both NAG and KIM-1 are within the normal range (healthy individuals) on the first post-transplant day and at the time of stabilisation. However, NGAL values on day 1 after transplantation are about six times higher than in healthy individuals, and decrease to the normal range at stabilisation time. It suggests that NGAL could be a more sensitive biomarker at the beginning of the transplant.

We have not found significant differences in the biomarker evolution of experimental groups, which could be due to the low “n” in living donor and double transplant groups, as well as the greater variability in cadaveric donors younger than 55 years old. Salazademh et al. [43] neither found differences when they studied NGAL excretion in transplanted patients from a living donor compared to those from a cadaveric donor.

Moreover, no major differences have been observed between the biomarker evolution expressed as urinary concentration and as daily excretion (Figure 2). Therefore, according to our results, it is not necessary to have urinary flow to obtain the prognostic information that NAG, NGAL, and KIM-1 could provide.

When analysing the relationship between urinary NAG and complications that occur in the immediate post-transplant period (Table 3), we observed that NAG has lower values on day 3 after transplant in patients who received a double kidney and pancreas transplant, and when cold ischemia time was less than 900 min (cold ischemia time of practically all patients who received this type of transplant). However, this result must be confirmed with a larger number of patients with double transplantation. Regarding the relationship between NAG and AR, our results do not find significant differences and are similar to others previously published [44,45]. Moreover, in some previous works [45,46] association was found between NAG and DGF. Although we have not found this association in this study, a previously described increase in NAG levels on day 3 after transplant (Figure 2) could indicate possible harmful events for the graft.

Regarding NGAL (Table 3), we have found a significant relation between this biomarker and donor age: specifically, NGAL on day 7 has higher values in patients with transplant from a cadaveric donor older than 55 years. It should be remembered that division into age is based on the delayed introduction of anticalcineurin inhibitors if the donor reached that age. To our knowledge, there is no relationship in the literature between donor age and urinary NGAL.

As in other previous publications [47,48], in our work we have not found a statistically significant association between NGAL and AR. However, this association has been referenced by Field et al. [49] in a study with different characteristics, since patients were sensitized, most were living donor transplants, and transplants were performed without group compatibility.

One of the most relevant results of our study is that high NGAL values during the first week after transplant are associated with a greater probability of suffering from DGF. This result is consistent with those of the meta-analysis published by Haase-Fielitz et al. [50] in which it was concluded that NGAL, measured in blood or/and urine after 6 or 12 post-transplant hours, correlates with DGF. Other subsequent publications have also correlated post-transplant NGAL elevation with DGF [51,52,53,54]. This fact could be very useful to adjust indication for renal biopsy, to program the visit interval, or to adjust immunosuppressive medication avoiding nephrotoxic drugs. NGAL has also been described as a predictive biomarker of kidney damage in other clinical circumstances, such as liver transplantation [55,56], tubulitis, or other tubular pathologies [57]. In addition, high NGAL levels one year after transplant have recently been associated with renal dysfunction [58], so this biomarker could also be useful in the long term. Another finding from our work is the correlation between urinary NGAL and the presence of AR and DGF simultaneously. However, this result seems to be due to the association between NGAL and DGF described above, and not to the combination of both events, since AR does not present a positive correlation with NGAL levels.

Despite the fact that various publications indicate that KIM-1 could be a good tool for the prognosis of kidney transplant viability [59,60,61,62], in our study, KIM-1 levels were not been related to the different post-transplant complications. Association between KIM-1 increase in donor and post-transplant renal function has also been described in [63]. However, some authors maintain that KIM-1 increase may be related to a protective effect on the kidney. Thus, in renal ischemia–reperfusion studies, KIM-1 expression persists during the first recovery week [37]. Therefore, the role of KIM-1 in a kidney transplant context is not clear and should be investigated [62].

Our results significantly show that patients with high urinary NAG or NGAL in the first week take longer to stabilise their renal function (Table 3). Regarding NGAL, this fact may be related to the greater DGF probability in these patients, a factor that determines renal function prognosis and recovery after transplantation [64]. This result is relevant as NGAL could provide prognostic information and be useful in avoiding treatments (such as immunosuppression regimens) that are potentially more damaging to the graft. However, the relationship between KIM-1 and stabilisation time is inverse: patients with lower KIM-1 values in the first week take longer to stabilise their renal function. This fact supports the idea reflected previously on the possible benefit of KIM-1 in post-transplant renoprotection [37,62].

One of the main limitations of our study is the lack of a sufficient number of biopsies to establish correlations between the biomarkers and the anatomopathological findings observed in the biopsy. Research in this sense is of great interest because by establishing these correlations, urinary biomarkers could mean avoiding having to perform many biopsies. While it is true that, in our study, AR was confirmed by biopsy.

In conclusion, our results show that the urinary biomarkers NAG, NGAL, and KIM-1 rise during the first post-transplant week; in addition, their concentrations/excretions during that first week correlate with some post-transplant events; it is worth noting that the relationship was found in the three biomarkers with renal function stabilisation time, since this event is probably influenced by others such as DGF or cold ischemia time. Therefore, our work provides evidence about the possible use of NAG, NGAL, and KIM-1 as prognostic tools for kidney transplantation. These results are a proof of concept and constitute a first step in the development of a clinical decision support system (CDSS) in the context of kidney transplant evolution. The second step will be to confirm and characterise the utility of each biomarker, and later it will be possible to propose the scientific-technological development of a rapid and precise diagnosis that allows the clinician to make decisions. The present work lays the foundations to carry out a broader analysis in terms of included biomarkers, combined analysis of biomarkers, number of patients, and post-transplant time studied.

## Figures and Tables

**Table 1:** Characteristics of the patients and transplants.

Type of Transplant	Living Donor	Cadaveric Donor (≥55 Years)	Cadaveric Donor (<55 Years)	Double Transplant (Kidney–Pancreas)	Total/ Media ± SEM
Patients number	9 (12.8%)	35 (50%)	17 (24.3%)	9 (12.8%)	70 (100%)
Sex of donor (F/M, number)	5/4 (56/44%)	25/10 (71/29%)	8/9 (47/53%)	2/7 (22/78%)	39/31 (56/44%)
Age of donor (years)	49.6 ± 3.8	66.6 ± 1.0	43.2 ± 2.6	35.0 ± 2.9	54.6 ± 1.8
Sex of recipient (F/M, number)	2/7 (22/78%)	13/22 (37/63%)	5/12 (29/71%)	4/5 (44/56%)	24/46 (34/66%)
Age of recipient (years)	46.6 ± 4.6	66.0 ± 1.3	47.0 ± 3.5	41.3 ± 2.0	56.0 ± 1.8
Cold ischemia time (minutes)	88 ± 4	934 ± 74	1120 ± 45	817 ± 48	855 ± 54
AR (Incidence)	1 (11%)	8 (23%)	3 (18%)	0 (0%)	12 (17%)
DGF (Incidence)	2 (22%)	16 (46%)	5 (29%)	0 (0%)	23 (33%)
AR + DGF (Incidence)	1 (11%)	6 (17%)	3 (18%)	0 (0%)	10 (14%)
Nephrotoxic drugs administration * (Incidence)	0 (0%)	8 (23%)	3 (18%)	2 (22%)	13 (19%)

SEM: standard error of the mean F: female. M: male. DGF: delayed graft function. AR: acute rejection. * Immunosuppressive drugs are excluded.

**Table 2:** Immunosuppressive drugs administered.

Immunosuppressive Drug	Posology	Observations
Basiliximab	20 mg, days 0 and 4	-Immunosuppression inducer; -In patients with double transplant (kidney-pancreas) it was replaced by thymoglobuline.
Tacrolimus	0.1 mg/kg, every 12 h	-If age of donor or recipient was greater than 55 years, its introduction was delayed until day 4; -It was replaced by CsA in one patient.
Mycophenolate Mofetil	1 g, every 12 h	-It was replaced by azathioprine in three patients.
6-Methylprednisolone	500 mg, day 1 125 mg, day 2	-Doses were halved for diabetic patients.
Prednisone	20 mg, from day 3, following a descending regimen up to 10 mg	-Doses were halved for diabetic patients.

**Table 3:** Relationship of NAG, NGAL, and KIM-1 with the main post-transplant events.

			Type of Transplant	AR	DGF	DGF and AR	Cold Ischemia Time	Nephrotoxic Drug Administration	Time to Renal Function Stabilisation
NAG	W1	χ^2^	8.708	2.737	3.109	4.178	4.514	2.064	39.427
		*p* value	0.191	0.254	0.211	0.653	0.341	0.356	<0.001
	D3	χ^2^	35.514	2.195	2.220	4.276	20.397	2.067	9.621
		*p* value	<0.001	0.334	0.329	0.639	<0.001	0.356	0.138
NGAL	W1	χ^2^	9.145	3.223	17.194	17.461	3.486	0.550	41.045
		*p* value	0.166	0.200	<0.001	0.008	0.480	0.760	<0.001
	D7	χ^2^	14.731	3.830	20.950	22.443	2.969	1.372	10.419
		*p* value	0.022	0.147	<0.001	0.001	0.563	0.504	0.108
KIM-1	W1	χ^2^	17.216	1.678	2.583	3.976	4.131	1.889	35.053
		*p* value	0.009	0.432	0.275	0.680	0.389	0.389	<0.001
	D3	χ^2^	35.726	1.183	4.436	6.390	14.341	3.291	3.107
		*p* value	<0.001	0.554	0.109	0.381	0.006	0.583	0.807

NAG: N-acetyl-d-glucosaminidase. NGAL: neutrophil gelatinase-associated lipocalin. KIM-1: kidney injury molecule-1. W1: highest biomarker concentration in the first post-transplant week. D3: biomarker concentration on day 3 after transplant. D7: biomarker concentration on day 7 after transplant. AR: acute rejection; DGF: delayed graft function. *p*: statistical significance according to Pearson’s χ^2^ test (statistically significant relationships are shaded in the table).

## Data Availability

The data presented in this study are available upon request from the corresponding author.

## Supplementary Materials

The following supporting information can be downloaded at: https://www.mdpi.com/article/10.3390/diagnostics13111843/s1, Table S1: Relationship between NAG on day 3 after transplant (NAG D3) and each transplant group; Table S2: Relationship between NAG on day 3 after transplant (NAG D3) and cold ischemia time of graft; Table S3: Relationship between the highest value of NAG in the first week after transplant (NAG W1) and stabilisation time of renal function; Table S4: Relationship between NGAL on day 7 after transplant (NGAL D7) and each transplant group; Table S5: Relationship between the highest value of NGAL in the first week after transplant (NGAL W1) and delayed graft function (DGF); Table S6: Relationship between NGAL on day 7 after transplant (NGAL D7) and delayed graft function (DGF); Table S7: Relationship between the highest value of NGAL in the first week after transplant (NGAL W1) and both delayed graft function (DGF) and acute rejection (AR); Table S8: Relationship between NGAL on day 7 after transplant (NGAL D7) and both delayed graft function (DGF) and acute rejection (AR); Table S9: Relationship between the highest value of NGAL in the first week after transplant (NGAL W1) and stabilisation time of renal function; Table S10: Relationship between the highest value of KIM-1 in the first week after transplant (KIM-1 W1) and each transplant group; Table S11: Relationship between KIM-1 on day 3 after transplant (KIM-1 D3) and each transplant group; Table S12: Relationship between KIM-1 on day 3 after transplant (KIM-1 D3) and cold ischemia time of graft; Table S13: Relationship between the highest value of KIM-1 in the first week after transplant (KIM-1 W1) and stabilisation time of renal function.
