# Supplementary material for: Evaluation of NAG, NGAL, and KIM-1 as Prognostic Markers of the Initial Evolution of Kidney Transplantation
Source: Diagnostics (Basel). 2023 May 25;13(11):1843. doi: 10.3390/diagnostics13111843 (PMC10252291; doi:10.3390/diagnostics13111843)
Supplement: Supplementary file 1 [file diagnostics-13-01843-s001.zip › diagnostics-2340840-supplementary.pdf]

Supplementary material

Table S1: Relationship between NAG on day 3 after transplant (NAG D3) and each transplant group: cadaveric donor under 55 years old (Cadaveric < 55), cadaveric donor over 55 years old (Cadaveric > 55), double kidney-pancreas transplant (Pancreas) and living donor (Living). Contingency table peformed by Pearson's χ^2 test. Missing: missing values. > 70 Perc: patients above the 70th percentile. < 70 Perc: patients below the 70th percentile.

			NAG D3			
			Missing	>70Perc	<70Perc	Total
Type of transplant	Cadaveric < 55	Observed frequency	0	4	13	17
		Expected frequency	2,0	4,7	10,3	17,0
	Cadaveric > 55	Observed frequency	2	11	22	35
		Expected frequency	4,1	9,6	21,3	35,0
	**Pancreas**	**Observed frequency**	6	2	**1**	9
		**Expected frequency**	1,0	2,5	**5,5**	9,0
	Living	Observed frequency	0	2	6	8
		Expected frequency	,9	2,2	4,9	8,0
Total		Observed frequency	8	19	42	69
		Expected frequency	8,0	19,0	42,0	69,0

Table S2: Relationship between NAG on day 3 after transplant (NAG D3) and cold ischemia time of graft. Contingency table peformed by Pearson's χ^2 test. Missing: missing values. > 70 Perc: patients above the 70th percentile. < 70 Perc: patients below the 70th percentile.

			NAG D3			
			Missing	>70Perc	<70Perc	Total
Cold ischemia	<120	Observed frequency	0	3	8	11
(minutes)		Expected frequency	1,3	3,0	6,7	11,0
	**120-900**	**Observed frequency**	6	7	**3**	16
		**Expected frequency**	1,9	4,4	**9,7**	16,0
	900-1440	Observed frequency	2	9	31	42
		Expected frequency	4,9	11,6	25,6	42,0
Total		Observed frequency	8	19	42	69
		Expected frequency	8,0	19,0	42,0	69,0

Table S3: Relationship between highest value of NAG in the first week after transplant (NAG W1) and stabilization time of renal function (based on plasma creatinine).. Contingency table peformed by Pearson's χ^2 test. Missing: missing values. > 70 Perc: patients above the 70th percentile. < 70 Perc: patients below the 70th percentile.

			NAG W1			
			Missing	>70Perc	<70Perc	Total
Stabilization time (days)	30-60	Observed frequency	0	15	32	47
		Expected frequency	,7	14,3	32,0	47,0
	60-90	Observed frequency	0	4	14	18
		Expected frequency	,3	5,5	12,3	18,0
	**90-120**	**Observed frequency**	0	**2**	**0**	2
		**Expected frequency**	,0	**,6**	**1,4**	2,0
Total		Observed frequency	1	21	47	69
		Expected frequency	1,0	21,0	47,0	69,0

Table S4: Relationship between NGAL on day 7 after transplant (NGAL D7) and each transplant group: cadaveric donor under 55 years old (Cadaveric < 55), cadaveric donor over 55 years old (Cadaveric > 55), double kidney-pancreas transplant (Pancreas) and living donor (Living). Contingency table peformed by Pearson's χ^2 test. Missing: missing values. > 70 Perc: patients above the 70th percentile. < 70 Perc: patients below the 70th percentile.

			NGAL D7			
			Missing	>70Perc	<70Perc	Total
Type of transplant	**Cadaveric < 55**	**Observed frequency**	1	2	**14**	17
		**Expected frequency**	2,5	4,2	**10,3**	17,0
	**Cadaveric > 55**	**Observed frequency**	3	**13**	19	35
		**Expected frequency**	5,1	**8,6**	21,3	35,0
	Pancreas	Observed frequency	3	0	6	9
		Expected frequency	1,3	2,2	5,5	9,0
	Living	Observed frequency	3	2	3	8
		Expected frequency	1,2	2,0	4,9	8,0
Total		Observed frequency	10	17	42	69
		Expected frequency	10,0	17,0	42,0	69,0

Table S5: Relationship between highest value of NGAL in the first week after transplant (NGAL W1) and delayed graft function (DGF). Contingency table peformed by Pearson's χ^2 test. Missing: missing values. > 70 Perc: patients above the 70th percentile. < 70 Perc: patients below the 70th percentile.

			NGAL W1			
			Missing	>70Perc	<70Perc	Total
**DGF**	**NO**	**Observed frequency**	1	**6**	**39**	46
		**Expected frequency**	,7	**13,3**	**32,0**	46,0
	**YES**	**Observed frequency**	0	**14**	**9**	23
		**Expected frequency**	,3	**6,7**	**16,0**	23,0
Total		Observed frequency	1	20	48	69
		Expected frequency	1,0	20,0	48,0	69,0

Table S6: Relationship between NGAL on day 7 after transplant (NGAL D7) and delayed graft function (DGF). Contingency table peformed by Pearson's χ^2 test. Missing: missing values. > 70 Perc: patients above the 70th percentile. < 70 Perc: patients below the 70th percentile.

			NGAL D7			
			Missing	>70Perc	<70Perc	Total
**DGF**	**NO**	**Observed frequency**	7	**7**	32	46
		**Expected frequency**	4,7	**12,0**	29,3	46,0
	**YES**	**Observed frequency**	0	**11**	12	23
		**Expected frequency**	2,3	**6,0**	14,7	23,0
Total		Observed frequency	7	18	44	69
		Expected frequency	7,0	18,0	44,0	69,0

Table S7: Relationship between highest value of NGAL in the first week after transplant (NGAL W1) and both delayed graft function (DGF) and acute rejection (AR). Contingency table peformed by Pearson's χ^2 test. Missing: missing values. > 70 Perc: patients above the 70th percentile. < 70 Perc: patients below the 70th percentile.

			NGAL W1			
			Missing	>70Perc	<70Perc	Total
**DGF/AR**	**NO/NO**	**Observed frequency**	1	**6**	**37**	44
		**Expected frequency**	,6	**12,8**	**30,6**	44,0
	NO/YES	Observed frequency	0	0	2	2
		Expected frequency	,0	,6	1,4	2,0
	**YES/NO**	**Observed frequency**	0	**8**	**5**	13
		**Expected frequency**	,2	**3,8**	**9,0**	13,0
	YES/YES	Observed frequency	0	6	4	10
		Expected frequency	,1	2,9	7,0	10,0
Total		Observed frequency	1	20	48	69
		Expected frequency	1,0	20,0	48,0	69,0

.

Table S8: Relationship between NGAL on day 7 after transplant (NGAL D7) and both delayed graft function (DGF) and and acute rejection (AR). Contingency table peformed by Pearson's χ^2 test. Missing: missing values. > 70 Perc: patients above the 70th percentile. < 70 Perc: patients below the 70th percentile.

			NGAL D7			
			Missing	>70Perc	<70Perc	Total
**DGF/AR**	**NO/NO**	**Observed frequency**	10	**4**	**30**	44
		**Expected frequency**	6,4	**10,8**	**26,8**	44,0
	NO/YES	Observed frequency	0	0	2	2
		Expected frequency	,3	,5	1,2	2,0
	**YES/NO**	**Observed frequency**	0	**8**	**5**	13
		**Expected frequency**	1,9	**3,2**	**7,9**	13,0
	YES/YES	Observed frequency	0	5	5	10
		Expected frequency	1,4	2,5	6,1	10,0
Total		Observed frequency	10	17	42	69
		Expected frequency	10,0	17,0	42,0	69,0

Table S9: Relationship between highest value of NGAL in the first week after transplant (NGAL W1) and stabilization time of renal function (based on plasma creatinine).. Contingency table peformed by Pearson's χ^2 test. Missing: missing values. > 70 Perc: patients above the 70th percentile. < 70 Perc: patients below the 70th percentile.

			NGAL W1			
			Missing	>70Perc	<70Perc	Total
Stabilization time	0-30	Observed frequency	1	1	0	2
(days)		Expected frequency	,0	,6	1,4	2,0
	30-60	Observed frequency	0	11	36	47
		Expected frequency	,7	13,6	32,7	47,0
	60-90	Observed frequency	0	6	12	18
		Expected frequency	,3	5,2	12,5	18,0
	**90-120**	**Observed frequency**	0	**2**	**0**	2
		**Expected frequency**	,0	**,6**	**1,4**	2,0
Total		Observed frequency	1	20	48	69
		Expected frequency	1,0	20,0	48,0	69,0

Table S10: Relationship between highest value of KIM-1 in the first week after transplant (KIM-1 W1) and each transplant group: cadaveric donor under 55 years old (Cadaveric < 55), cadaveric donor over 55 years old (Cadaveric > 55), double kidney-pancreas transplant (Pancreas) and living donor (Living). Contingency table peformed by Pearson's χ^2 test. Missing: missing values. > 70 Perc: patients above the 70th percentile. < 70 Perc: patients below the 70th percentile.

			KIM-1 W1			
			Missing	>70Perc	<70Perc	Total
Type of transplant	**Cadaveric < 55**	**Observed frequency**	**0**	**8**	**9**	17
		**Expected frequency**	**,2**	**3,4**	**13,3**	17,0
	Cadaveric > 55	Observed frequency	0	5	30	35
		Expected frequency	,5	7,1	27,4	35,0
	Pancreas	Observed frequency	1	0	8	9
		Expected frequency	,1	1,8	7,0	9,0
	Living	Observed frequency	0	1	7	8
		Expected frequency	,1	1,6	6,3	8,0
Total		Observed frequency	1	14	54	69
		Expected frequency	1,0	14,0	54,0	69,0

Table S11: Relationship between KIM-1 on day 3 after transplant (KIM-1 D3) and each transplant group: cadaveric donor under 55 years old (Cadaveric < 55), cadaveric donor over 55 years old (Cadaveric > 55), double kidney-pancreas transplant (Pancreas) and living donor (Living). Contingency table peformed by Pearson's χ^2 test. Missing: missing values. > 70 Perc: patients above the 70th percentile. < 70 Perc: patients below the 70th percentile.

			KIM-1 D3			
			Missing	>70Perc	<70Perc	Total
Type of transplant	**Cadaveric < 55**	**Observed frequency**	0	**7**	10	17
		**Expected frequency**	2,0	**3,7**	11,3	17,0
	**Cadaveric > 55**	**Observed frequency**	1	6	**28**	35
		**Expected frequency**	4,1	7,6	**23,3**	35,0
	Pancreas	Observed frequency	6	0	3	9
		Expected frequency	1,0	2,0	6,0	9,0
	Living	Observed frequency	1	2	5	8
		Expected frequency	,9	1,7	5,3	8,0
Total		Observed frequency	8	15	46	69
		Expected frequency	8,0	15,0	46,0	69,0

Table S12: Relationship between KIM-1 on day 3 after transplant (KIM-1 D3) and cold ischemia time of graft. Contingency table peformed by Pearson's χ^2 test. Missing: missing values. > 70 Perc: patients above the 70th percentile. < 70 Perc: patients below the 70th percentile.

			KIM-1 D3			
			Missing	>70Perc	<70Perc	Total
Cold ischemia	<120	Observed frequency	1	2	8	11
(minutes)		Expected frequency	1,3	2,4	7,3	11,0
	**120-900**	**Observed frequency**	6	2	**8**	16
		**Expected frequency**	1,9	3,5	**10,7**	16,0
	900-1440	Observed frequency	1	11	30	42
		Expected frequency	4,9	9,1	28,0	42,0
Total		Observed frequency	8	15	46	69
		Expected frequency	8,0	15,0	46,0	69,0

Table S13: Relationship between highest value of KIM-1 in the first week after transplant (KIM-1 W1) and stabilization time of renal function (based on plasma creatinine).. Contingency table peformed by Pearson's χ^2 test. Missing: missing values. > 70 Perc: patients above the 70th percentile. < 70 Perc: patients below the 70th percentile.

			KIM-1 S1			
			Missing	>70Perc	<70Perc	Total
Stabilization time	0-30	Observed frequency	1	0	1	2
(days)		Expected frequency	,0	,4	1,6	2,0
	**30-60**	**Observed frequency**	0	**11**	36	47
		**Expected frequency**	,7	**9,5**	36,8	47,0
	60-90	Observed frequency	0	3	15	18
		Expected frequency	,3	3,7	14,1	18,0
	90-120	Observed frequency	0	0	2	2
		Expected frequency	,0	,4	1,6	2,0
Total		Observed frequency	1	14	54	69
		Expected frequency	1,0	14,0	54,0	69,0
